# Knowledge-based patient screening for rare and emerging infectious/parasitic diseases: a case study of brucellosis and murine typhus.

**DOI:** 10.3201/eid0301.970111

**Published:** 1997

**Authors:** C N Carter, N C Ronald, J H Steele, E Young, J P Taylor, L H Russell, A K Eugster, J E West

**Affiliations:** Texas A&M University, College Station, USA.

## Abstract

Many infectious and parasitic diseases, especially those newly emerging or reemerging, present a difficult diagnostic challenge because of their obscurity and low incidence. Important clues that could lead to an initial diagnosis are often overlooked, misinterpreted, not linked to a disease, or disregarded. We constructed a computer-based decision support system containing 223 infectious and parasitic diseases and used it to conduct a historical intervention study based on field investigation records of 200 cases of human brucellosis and 96 cases of murine typhus that occurred in Texas from 1980 through 1989. Knowledge-based screening showed that the average number of days from the initial patient visit to the time of correct diagnosis was significantly reduced (brucellosis-from 17.9 to 4.5 days, p = 0.0001, murine typhus-from 11.5 to 8.6 days, p = 0.001). This study demonstrates the potential value of knowledge-based patient screening for rare infectious and parasitic diseases.


### Knowledge-Based Patient Screening for Rare and Emerging Infectious/Parasitic Diseases: A Case Study of Brucellosis and Murine Typhus

Many infectious and parasitic diseases, especially those newly emerging or reemerging, present a difficult diagnostic challenge because of their obscurity and low incidence. Important clues that could lead to an initial diagnosis are often overlooked, misinterpreted, not linked to a disease, or disregarded. We constructed a computer-based decision support system containing 223 infectious and parasitic diseases and used it to conduct a historical intervention study based on field investigation records of 200 cases of human brucellosis and 96 cases of murine typhus that occurred in Texas from 1980 through 1989. Knowledge-based screening showed that the average number of days from the initial patient visit to the time of correct diagnosis was significantly reduced (brucellosis—from 17.9 to 4.5 days, $p = 0.0001$, murine typhus—from 11.5 to 8.6 days, $p = 0.001$). This study demonstrates the potential value of knowledge-based patient screening for rare infectious and parasitic diseases.

Knowledge-based medical decision support systems allow the user to relate clinical and laboratory manifestations from which a list of possible diagnoses can be generated. Studies have demonstrated that such a list is extremely useful for alerting clinicians and epidemiologists to diseases seen rarely, if ever, and to unusual clinical manifestations of diseases (1,2).

This report describes the development and laboratory testing of a medical decision support system designed to facilitate early recognition of rare and unusual infectious and parasitic diseases. The assumption is made that early diagnosis can improve the course of treatment as well as the overall health of the patient.

### Constructing a Knowledge Base

In this study, the knowledge base was restricted to the 223 diseases common to humans and animals. Several disease information axes were identified for inclusion in the medical knowledge base. For each disease, nomenclature along with any recognized synonyms, etiology, epidemiologic descriptions, diagnostic procedures, diagnostic procedure findings, and clinical signs and symptoms were gathered and abstracted from recent editions of standard medical texts. In addition, occupational risks, food consumption data, travel history, and animal/insect exposure were included. Finally, basic treatment, management, and prevention protocols were also recorded (3-10). Bibliographic citations for all entries into the knowledge base were tied to each information axis to authenticate the content and provide a quick reference for additional reading. Glossaries of knowledge base entries (e.g., signs, symptoms, occupation, diagnostic procedure results, geography, animal/insect exposure) to be used in generating a differential diagnosis were created to ensure the consistency of the lexicon. Audits were performed to validate the knowledge base entries against the glossaries. The resulting knowledge base contains the entries listed in Table 1. A batch

Table 1. Knowledge base of 223 diseases at the time of case study

| Information axis | Disease entities | Lines of text |
|---|---|---|
| Etiology | 410 | |
| Epidemiology[a] | | 9724 |
| Immunodeficiency info | | 352 |
| Diagnostic procedures | 306 | |
| Diagnostic procedure | 637 | |
| Occupational links[b] | 37 | |
| Geographic links (countries)[c] | 191 | |
| Vectors/Invertebrate hosts | 703 | |
| Symptoms/Clinical signs | 1422 | |
| Treatment/Prevention | | 3162 |
| Special considerations[d] | | 308 |
| Bibliographic citations | 838 | |
| Descriptive notes[e] | 322 | |

[a]Includes specific etiologic information, reservoirs, portal of entry/escape, mode of transmission/dissemination, susceptible hosts, incubation period, seasonal distribution, age/sex/race/religion/occupation relationships, mortality and zoonotic classification.
[b]Lists occupations with increased potential for exposure.
[c]Lists countries where the disease is known to occur.
[d]Describes additional disease attributes not fitting other categories.
[e]Pop-up definitions of medical terms and concepts in the knowledge base.

procedure indexing all findings to diseases for use by the inference engine resulted in more than 12,000 finding/disease links. The inference engine is the portion of the decision support system used to derive useful clinical information (e.g., possible diagnoses). It is composed of nine program modules containing more than 10,000 lines of C language code linked into one executable program.

### Differential Diagnosis Methods

The goal of the system is to remind the user of relationships between clinical signs, symptoms, and findings and the diseases themselves. Therefore, a simple, intuitive pattern-matching technique was employed. The user sets a "closeness-of-fit" parameter in the system that determines how tight the relationship between case signs/findings and diseases must be before a disease can become a candidate for the differential diagnosis list. A simple ratio is created with signs/findings entered that fit each disease as the numerator and the total number of signs/findings entered as the denominator. The ratio ranges from zero to 1.00 with 1.00 implying a perfect fit. If this value is equal to or greater than the "closeness-of-fit" parameter set by the user (also ranging from zero to 1.00), the disease is entered in the differential diagnosis list as a candidate. The ratio for each disease shows how close the clinical manifestations are to previously published descriptions of the disease. For example, if seven clinical signs and findings are collected on a case and six fit a particular disease by the pattern-matching algorithm, the case fit would be 0.86. If the case fit parameter in the system is set to 0.86 or greater, that disease would be added to the differential diagnosis list. If the case fit parameter is set to 1.00, only diseases that fit the clinical manifestations perfectly would become differential diagnosis candidates. Signs and findings are not weighted by the system.

### Decision Support System Trial on Murine Typhus Cases

The purpose of this trial was to determine if the intervention of the support system at the time of the first physician visit would significantly alter the mean number of days to the correct diagnosis. Investigation records of 96 cases of murine typhus occurring in Texas from 1979 to 1987 were obtained from the Texas Department of Health. All records were complete and were used in the study. Each record contained the date of the first contact with a physician (dp). The date on which the indirect fluorescence antibody test for *Rickettsia typhi* was ordered was defined as the date the correct diagnosis was suspected (ds). Therefore, the number of days from the first contact with a physician (dp) to the correct diagnosis without decision support system intervention ($dc_{ni}$) was defined as follows: $dc_{ni}$=ds-dp.

The number of days till the correct diagnosis was suspected ($dc_{ni}$) was calculated for all cases without support system intervention. A case was classified as missed if $dc_{ni}$ >13 days. This number was derived from the worst-case scenario for a murine typhus test turnaround from the Texas Department of Health. During 1979-1987, the department had the only laboratory in Texas that used the indirect fluorescence antibody test for confirming murine typhus. On the basis of this criterion, 23 of the 96 original cases fell into the missed category.

The clinical history, symptoms, physical findings, and some preliminary laboratory findings as noted in the investigation records for the 23 missed patients were entered in the support system and processed at case fit parameter values of 0.25, 0.50, 0.75, and 1.00. Specific historical data such as occupation, travel, and animal/insect exposure, if available, were also entered in the support system. The support system was said to have suggested the correct diagnosis of murine typhus if the disease was included in a differential diagnosis list of five or fewer diseases at a case fit parameter of 0.50 or greater.

Testing for murine typhus was assumed to be ordered immediately if the disease was suggested by the support system. This effectively set the number of days to suspect the correct diagnosis with intervention ($dc_{wi}$) to 1 day under these conditions.

The distributions of $dc_{ni}$ and $dc_{wi}$ were found to be Gaussian (PROC UNIVARIATE, SAS). Therefore, parametric statistical techniques were used for data analysis. A paired t-test was run on $\mu_{ni}$ and $\mu_{wi}$ (mean time to suspecting murine typhus with and without the system, respectively) to see if there was a significant difference in the means (PROC MEANS, SAS) (14).

In 11 (48%) of the 23 cases defined as missed, murine typhus was clearly suggested by the support system in a differential list of five or fewer possibilities by using only clinical history, signs, symptoms, and preliminary laboratory data if available. In six (26%) of these cases, murine typhus was the only disease suggested by the support system. The support system did not suggest the correct disease in 12 (52%) of the cases classified as missed. Table 2 lists the most common diseases that also appeared in the differential diagnosis lists for murine typhus cases.

Table 2. Other diseases commonly listed in the differential diagnosis of murine typhus

| | |
|---|---|
| 1. Lyme borreliosis | 7. Relapsing fever |
| 2. Tularemia | 8. Avian chlamydiosis |
| 3. Brucellosis | 9. Leptospirosis |
| 4. Zoonotic epidemic typhus | 10. Salmonellosis |
| 5. Rocky Mountain spotted fever | 11. Babesiosis |
| | 12. Malaria |
| 6. Rat bite fever | 13. Q fever |

The mean number of days to suspect the correct diagnosis without support system intervention ($\mu_{wi}$) was 11.5 days. The mean number of days to suspect the correct diagnosis with support system intervention ($\mu_{wi}$) was 8.6 days, an improvement of 2.9 days ($p$ = 0.001).

### Decision Support System Trial on Brucellosis Cases

Investigation records of 342 cases of brucellosis occurring in Texas from 1980 to 1989 were also obtained from the Texas Department of Health. Of these records, 13 were incomplete, and 129 involved cases of recrudescence of the disease or had no specific date of onset of illness and were excluded from the study. The remaining 200 records contained the date of onset of illness. However, the day of the first physician visit was not available and had to be extrapolated from the day of onset of illness. The average number of days from onset of illness to the first physician visit was assumed to be 4 days, on the basis of a study in Texas for patients with murine typhus (12).

The day that the correct diagnosis was suspected was defined as the day on which the serum agglutination test or a bacterial culture for brucellosis was ordered. The number of days from the first contact with a physician to the day the correct diagnosis was suspected without support system intervention ($dc_{ni}$) was calculated as indicated above.

A case was classified as missed if $dc_{ni}$ >11 days. This number was derived from the worst-case scenario for a brucellosis test turnaround from the Texas Department of Health. By this liberal criterion, 98 of the 200 original cases still fell into the missed category.

The clinical history, symptoms, physical findings, and some preliminary laboratory findings as noted in the investigation records for the 98 missed patients were entered in the support system and processed at case fit parameter values of 0.25, 0.50, 0.75, and 1.00. Specific historical data such as occupation, travel, and animal/insect exposure, if available, were also entered in the support system. By definition, the system gave the correct diagnosis of brucellosis if the disease was included in a differential diagnosis list of five or fewer diseases at a case fit parameter of 0.50 or greater.

It was assumed that the testing for brucellosis would be ordered immediately if the disease was suggested by the support system. This effectively sets the number of days to suspect the correct diagnosis with intervention ($dc_{wi}$) to 1 day under these conditions. The distributions of $dc_{ni}$ and $dc_{wi}$ were also Gaussian and analyzed by paired t-tests on $\mu_{ni}$ and $\mu_{wi}$ (14).

In 86 (88%) of the 98 cases defined as missed, brucellosis was clearly suggested by the support system in a differential list of five or fewer possibilities by using only clinical history, signs, symptoms, and preliminary laboratory data if available. In 69 (70%) of the cases, brucellosis was the only disease suggested. The support system did not suggest the correct disease in only 12 (12%) of the cases classified as missed. Table 3 lists the most common diseases that also appeared in the differential diagnosis of brucellosis.

Table 3. Other diseases commonly listed in the differential diagnosis of brucellosis

| | |
|---|---|
| 1. Toxoplasmosis | 6. Rat bite fever |
| 2. Q fever | 7. Listeriosis |
| 3. Viral hepatitis A | 8. Blastomycosis |
| 4. Salmonellosis | 9. Trichinellosis |
| 5. Histoplasmosis | 10. Lyme borreliosis |

The mean number of days to suspect the correct diagnosis without support system intervention ($\mu_{ni}$) was 17.9 days. The mean number of days to suspect the correct diagnosis with support system intervention was 4.5 days, an improvement of 12.9 days ($p$ = 0.0001). However, data were derived by using an extrapolated day of first physician visit, and that may have affected these comparisons.

The brucellosis investigation records screened showed that of nine cases, three involved apparently healthy pregnancies, one a premature delivery, one a miscarriage, one seizures, one weakness and chronic headaches, and two deaths. In eight of the nine cases, the support system correctly suggested brucellosis. In addition, 59 (30%) of the patients with brucellosis were hospitalized, some for extended periods during the diagnostic phase.

Twenty-one of 23 murine typhus patients were hospitalized, some for extended periods during the diagnostic phase of the case. Furthermore, many patients had to undergo expensive diagnostic testing, including 19 electrocardiograms, four computer-aided tomography scans, and a various other procedures such as bone scans, liver scans, barium enemas, and renal and pelvic sonograms. Earlier diagnoses of brucellosis and murine typhus could have eliminated the need for many of the diagnostic procedures, shortened hospital stays, and improved the course of treatment.

On the average, it took approximately 3 minutes of interaction with the decision support system to construct a differential diagnosis. Therefore, this type of screening could easily become part of the routine history-taking and physical exam of a patient. Furthermore, this study underlines the discriminatory power of the clinical history and physical signs and symptoms in suspecting the presence of a certain disease in a patient. Factors such as the patient's occupation, travel history, animal/insect exposure, and unusual dietary habits can be especially important in helping to diagnose many rare infectious and parasitic illnesses.

The results of this evaluation indicate that for two rare diseases (brucellosis and murine typhus), the decision support system appears to perform well (i.e., with high sensitivity) in suggesting the correct diagnosis in a patient. However, the specificity of the system has not been evaluated. A prospective double-blinded study in a general clinical or hospital setting would make that determination.

**Craig N. Carter,* Norman C. Ronald,† James H. Steele,‡ Ed Young,§ Jeffery P. Taylor,¶ Leon H. Russell, Jr.,* A. K. Eugster,* Joe E. West***

*Texas A&M University, College Station, Texas, USA; †Texas Medical Informatics, Inc., College Station, Texas, USA; ‡University of Texas School of Public Health, Houston, Texas, USA; §Veteran Affairs Medical Center, Houston, Texas, USA; and ¶Texas Department of Health, Austin, Texas, USA

### References

1. Elkin PL, Barnett GO, Famigletti KT, Kim RJ. Closing the loop on diagnostic decision support systems. Proc Annu Symp Comput Appl Med Care, 1990;589-93.
2. Johnston ME, Langton KB, Haynes RB, Mathieu A. Effects of computer-based clinical decision support systems on clinician performance and patient outcome. Yearbook of Medical Informatics 1995:486-93.
3. Acha PN, Szyfres B. Zoonoses and communicable diseases common to animals and man. 2nd ed. Washington (DC): Pan American Health Organization, 1987.
4. Steele JH. CRC handbook series in zoonoses. 1st ed. Boca Raton (FL): CRC Press, 1981.
5. Young EJ. Human brucellosis. Review of Infectious Diseases 1983;5:821-41.
6. Benenson AS. Control of communicable diseases in man. 15th ed. Washington (DC): American Public Health Association, 1990.
7. Mandell GL, Douglas RG, Bennett JE. Principles and practice of infectious diseases 3rd ed. New York: Churchill Livingstone, 1990.
8. Greene CE. Infectious diseases of the dog and cat. 1st ed. Philadelphia: Saunders, 1990.
9. Blood DC, Radostits OM. Veterinary medicine. 7th ed. London: Billiere Tindall, 1989.
10. Smith BP. Large animal internal medicine. 1st ed. St. Louis: Mosby, 1990.
11. Hughes, C. The representation of uncertainty in medical expert systems. Med Inf 1989;14:269-79.
12. Dumler JS, Taylor JP, Walker DH. Clinical and laboratory features of murine typhus in Texas, 1980-1987. JAMA 1991;266:1365-70.
13. Buck C, Donner A. The design of controlled experiments in the evaluation of non-therapeutic interventions. Journal of Chronic Diseases 1982;35:531-8.
14. Ott L. An introduction to statistical methods and data analysis 3rd ed. Boston: PWS-Kent Publishing, 1988.
